# Quantifying the daily intake of water from morning and spot urine samples; retrospective analysis of a clinical trial in volunteers

**DOI:** 10.1186/s40795-022-00660-2

**Published:** 2023-01-02

**Authors:** Robert G. Hahn

**Affiliations:** 1 Research Unit, Södertälje Hospital, Södertälje, Sweden; 2 Karolinska Institutet at Danderyds Hospital (KIDS), 152 86 Södertälje, Sweden

**Keywords:** Biomarkers, Creatinine, Hydration status, Fluid retention, Osmolality, Urine analysis, ROC, Area under the curve

## Abstract

**Background:**

The hydration status can be indicated by biomarkers in the urine. However, the sensitivity and specificity of single measurements of biomarkers in morning urine and spot urine samples to quantify previous and current daily water ingestion is unclear.

**Methods:**

The water content of food and liquid consumed by 20 volunteers (mean age 42 years) was calculated daily for two weeks. The volunteers increased their consumption of water by approximately 30% during the second week. They measured their excreted urine volume and sampled the morning urine and 24-h collections of urine for analysis of osmolality and creatinine during the first four days of both weeks (*N* = 157). The same biomarkers of hydration were measured in spot samples taken at every voiding on the other days (*N* = 762). Receiver operating characteristic (ROC) curves were used to study the ability of pre-specified ranges of biomarkers to quantify the water intake.

**Results:**

The biomarkers in the morning urine obtained during normal fluid intake quantified the water consumption with an average area under the ROC curve (AUC) of 0.72 for osmolality and 0.66 for creatinine. Spot urine yielded an AUC of 0.74 for osmolality and 0.70 for creatinine. The AUCs obtained for days of increased fluid intake were approximately 10% lower. Large intakes (3–4 L daily) were identified with a sensitivity of 50–80% and low intakes (< 1.5–2 L) with a sensitivity of 20–50%, while false positives occurred in approximately 10%.

**Conclusion:**

Biomarkers in morning urine and spot urine samples distinguished between large and small daily water intakes. Osmolality was slightly superior to creatinine. The indications were less useful during days of increased fluid intake.

**Supplementary Information:**

The online version contains supplementary material available at 10.1186/s40795-022-00660-2.

## Introduction

Adequate intake of water is essential to maintain health and is therefore of interest to assess in population studies and healthcare. However, the daily ingestion of water is cumbersome to quantify with confidence. Uncertainty often arises about how trustful self-reported assessments are and how to estimate the water content of solid food [1].

A simple way to evaluate dehydration in a graded fashion is to analyze the concentration of metabolic waste products in the urine, such as osmolality and creatinine [2–4]. Waste products are excreted at a stable rate throughout the day regardless of fluctuations in urine flow rate. Higher concentrations are measured if the urinary excretion is small. These biomarkers of hydration show great variation in the general population [5] which has been clearly linked to inter-individual differences in the habitual intake of water [6, 7]. However, how well a single measurement of metabolic waste products reflects the water intake is unclear.

The time of day when the sample should be taken to reflect the fluid balance has been a matter of controversy. Measuring biomarkers in urine that has been collected over 24 h is likely to accurately reflect the 24-h intake of water. By contrast, the morning urine is affected by the kidney´s night-time antidiuresis and spot samples at daytime may be confounded (diluted) by recent intake of water and food. Researchers have also sampled urine at well-defined times of the day, albeit not in close conjunction with intake of food and drink [7, 8]. Bottin et al*.* reported that urine osmolality and urine-specific gravity in spot samples collected between 14–20 h correlates closely with measurements of these biomarkers in 24-h collections of urine [8]. Spot sampling taken at any time of the day without consideration of food and water ingestion would be the most practical approach, but its value has been seriously questioned and frankly discarded on theoretical grounds [9].

The present report evaluates the sensitivity and specificity of single measurements of biomarkers in morning urine and randomly taken spot urine samples used to quantify previous and current daily water ingestion in volunteers as given by weighed food and measured fluid collections. The hypothesis was that the morning urine readily predicts the daily intake of water on the day before the sample is taken, while randomly taken spot samples reasonably well predicts the water consumption on the same day.

## Methods

### Approvals

Data were collected between November 2016 and March 2017 from 20 volunteers who recorded all intakes of food and water for two weeks.

The study followed the guidelines of the Declaration of Helsinki, and the protocol was approved by the Regional Ethics Committee of Stockholm on 15/06/2016 (Dnr. 2016/826–31, Chairperson Hans Glaumann) and registered in a database, http://www.isrctn.com, as identifier ISRCTN12215472 on 18/11/2016. Written and verbal consent was obtained from all participants. Other data from the study have been reported elsewhere [10]. The presentation follows the CONSORT checklist.

### Screening

The participants in the fluid intake and urine sampling study were selected after a screening procedure of 150 hospital staff workers. The screening study was advertised on the local Internet system and open to all staff. Exclusion criteria were professional sports activities and disease that required daily medication or a special diet. Enrolment was stopped when 150 participants had been recruited. Written informed consent was obtained from each volunteer. They were given a plastic tube identical to the ones used for urine sampling in the hospital and self-delivered a fresh 10 mL spot urine sample and a health status self-examination questionnaire at the Research Unit. The participants were not allowed to ingest any fluid within 2 h prior to voiding. The degree of urine concentration was assessed by measuring the urine-specific gravity on a Clinitek Status® Analyser (Siemens Healthcare Diagnostics) immediately and in the same room as the urine sample was delivered. Thereafter, the samples were discarded. When the screening procedure was completed, the volunteers with the highest and lowest urine-specific gravity were offered to participate in the subsequent fluid intake and urine sampling study which was ended when 20 participants had been enrolled. New oral and written information was given to these 20 volunteers, and they underwent a formal health examination to confirm that they were in good health.

### Fluid intake

The study started at 7 am on Day 1 and ended at 7 am on Day 15. On all these days, the volunteers weighed all ingested liquids and foodstuffs on a scale, recorded the type of food, and recorded the time for the meal in a protocol. The water and nutritional contents were calculated by a dietitian using the Dietist Net software (Kost och Näringsdata, Bromma, Sweden; available in English at http://www.kostdata.se). The water intakes included both ingested liquids and the water contents of the food.

During the first week, the 20 volunteers ingested liquid as usual. They were instructed to ingest one more glass of water with each meal during the second week.

### Urine sampling

On Days 1–4 and 8–11, the volunteers collected all urine and measured the volume in the standard plastic bucket used for this purpose in our hospital. These buckets are graded to the nearest 50 mL. Urine collection started at 7 am and ended 7 am the following day.

The participants had received a urine collection kit and delivered two samples taken from each of the 24 h urine collections for biomarker analysis at the hospital´s clinical chemistry laboratory ("24 h urine collection"). They also provided two samples of the first portion of urine voided each morning before any fluid or food was ingested on that day ("morning urine").

On six days (on Days 4–7 and 11–15), the volunteers did not measure the urine volume but instead provided a spot urine sample every time they voided ("spot samples"). The urine was not collected at fixed times but whenever the volunteer felt the urge to void.

### Biochemical analyses

The urine osmolality and the urinary creatinine concentration were used as biomarkers of the degree of renal water conservation. The participants, who were all hospital staff, delivered the urine samples to the clinical chemistry by themselves in the morning, usually just before starting to work, and  also on weekends. Both were stored in a refrigerator at a temperature of + 4° C until analysis performed within 36 h after sampling at the certified clinical chemistry laboratory at Karolinska University Hospital in Stockholm. Urine osmolality was measured based on freezing-point depression with an Advanced 2020 osmometer (Molek AB, Sweden). Urine creatinine was measured and Cobas 8000 analyzer (Roche Diagnostics, Basel, Switzerland) which uses a colorimetric assay by adding picric acid to the sample, which thereby combined with creatinine to form a yellow-orange complex (Jaffé method). The coefficient of variation was 3% for osmolality and 5% for creatinine (at 6 mmol/L) [5]. The analytical precision was guaranteed provided that the analysis was performed within 36 h.

### Design

The study was longitudinal single-group clinical trial with one intervention, which consisted in increasing the fluid intake during the second week. The participants were aware of their own food and water intakes and their measured urine volumes but not of the results of the biomarker measurements. The researcher (RGH) was only aware of the results of the screening procedure and health examination until the study was completed.

### Statistics

Continuous data are presented as the mean and standard deviation (SD). Normal distribution was confirmed by the Kolmogorov–Smirnov´s test. Differences between subgroups were studied by one-way ANOVA followed by the Scheffé post hoc test. Receiver operating characteristic (ROC) curves were used to express the ability of arbitrary ranges of biomarkers to predict the water intake and the excreted urine. For example, all fluid intakes linked to urine osmolality between 50 and 400 mosmol/kg were set to be 1 and all fluid intakes linked to urine osmolality > 400 were set to 0. The ROC curve then showed the probability of how well urine osmolality of 50–400 indicates gradually changing fluid intakes. The same procedure was repeated for other ranges of biomarkers. ROC curves plot sensitivity (true positive fraction) *versus* 1 – specificity (false positive fraction) and gives the calculated area under the curve (AUC) for this relationship, which reflects how well ranges of fluid intake can be separated. The prediction given by the ROC curve is statistically significant if the 95% confidence interval does not include 0.5. Correlations between water intake (independent variable) and the urinary biomarkers of hydration (dependent variables) were also studied by simple linear regression analysis where *r* = correlation coefficient. 

The original prospective study was powered (95%) to detect a change in urine osmolality of 200 mosmol/kg on the *P* < 0.05 level when the water intake was increased during the second week [9]. A post hoc power analysis for the present analysis was based on the ROC curves, which show SD of approximately 5% for the morning urine. A difference in AUC of 4% between the ability of the morning and spot urine samples to detect ranges of water ingestion could then be disclosed with a power of 92% (*N* = 20, *P* < 0.05, paired sampling).

The statistical software used was SPSS version 28.0.0 for Mac (IBM Corp., Armonk, NY) while the software used for the power calculation was GPower version 3.1.9.2 (freeware from Universität Dusseldorf, Germany). *P* < 0.05 was considered statistically significant.

## Results

The 20 volunteers (16 women and 4 men) who agreed to participate in the fluid balance study had a mean age of 42 years (SD, 11; range 23–62) and weighed 73 kg (SD 11, range 56–94).

### 24-h collections of urine

All 20 volunteers should deliver a 24-h collection of urine during each of the first 4 days of each week, but an incomplete report from one volunteer reduced the number of evaluable collections from 160 to 157.

The ROC curves for the urinary excretion of osmotically active substances and creatinine measured in these 24 h collections of urine showed an average AUC of 0.88, which then reflects how well the ranges of urine volumes could be separated by the biomarkers (Table 1, upper section). The AUC averaged 0.77 when the biomarkers were used to predict the water intake during the same day (Table 1, lower section).

**Table 1:** Area under the curve (AUC) and 95% confidence interval (CI) of ROC curves when ranges of urine osmolality and urinary creatinine measured on a 24-h collection of urine are used to predict the 24 h urine volume and the 24 h intake of water. All calculations were based on 157 urine collections performed during both normal and increased water consumption

Volume (L)	Osmolality 50–400	Osmolality 50–600	Osmolality > 800	Creatinine 2–6	Creatinine 2–9	Creatinine > 15
PREDICTION OF URINE VOLUME—analysis of 24 h urine collection						
AUC (95% CI)	0.87 (0.82–0.93)	0.87 (0.81–0.93)	0.89 (0.81–0.97)	0.86 (0.80–0.93)	0.84 (0.78–0.90)	0.92 (0.82–1.00)
Pos./neg	58 / 99	115 / 42	20 / 137	66 / 91	96 / 61	12 / 145
PREDICTION OF WATER INTAKE—analysis of 24-h urine collection						
AUC (95% CI)	0.77 (0.70–0.85)	0.77 (0.69–0.85)	0.74 (0.63–0.85)	0.75 (0.67–0.83)	0.75 (0.67–0.82)	0.81 (0.71–0.90)
Pos./neg	58 / 99	115 / 42	20 / 137	66 / 91	96 / 61	12 / 145

*Pos./neg.* Number of positive and negative indications, *AUC* Area under the curve, *95% CI* 95% confidence interval

### Morning urine

**Fig. 1:**
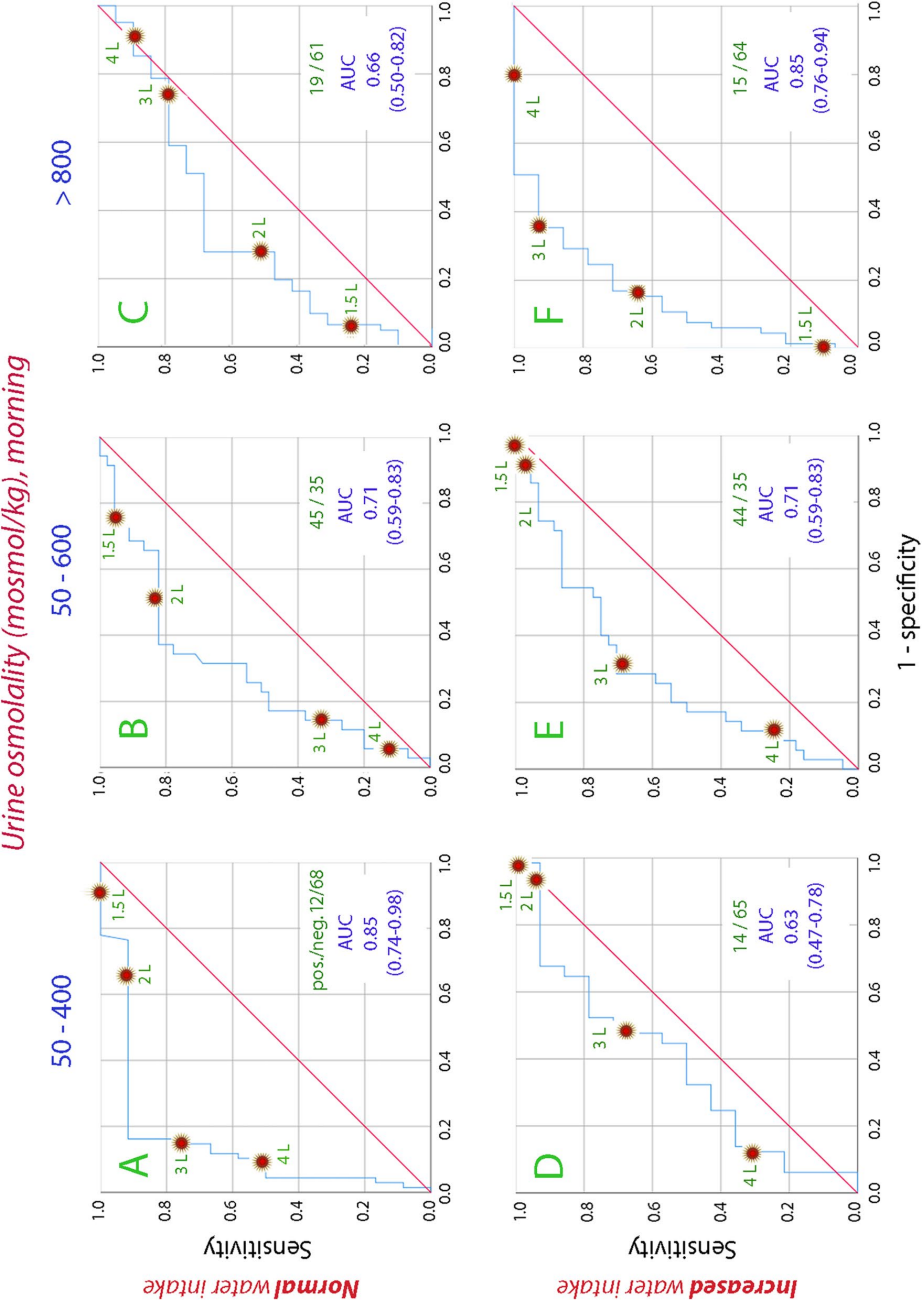
Receiver operating characteristic (ROC) curves that show the sensitivity and (1 – specificity) for ranges of urine osmolality in the morning urine to indicate the water consumption during the preceding day. AUC = area under the curve. The 20 volunteers delivered data on 4 consecutive days during normal fluid intake and 4 consecutive days with increased water intake. One measurement from the second period was missing

Biomarkers measured in the morning urine (i.e., the first void before any intake of water) were used to estimate the intake of water during the *preceding* 24 h. Separation was made for the four days of normal fluid intake (Days 1–4) and the four days of increased fluid intake (Days 8–11). Here, the AUC averaged 0.72 for osmolality (Fig. 1) and 0.66 for creatinine (Supplementary Fig. 1).

Low urine osmolality was most useful in predicting a large water intake during the period of normal water intake. For example, ingestion of > 3 L per 24 h was indicated with a sensitivity of 0.75 and a false positive fraction (i.e., 1 – specificity) of only 0.18 (Fig. 1A).

At the other end, high osmolality indicated daily fluid intake of < 3 L during the period of increased fluid intake with sensitivity of 0.93 and (1 – specificity) of 0.38. Intakes of < 2 L were too few to be evaluated, which was due to the protocol-based increase in daily water consumption during the second week (Fig. 1F).

### Spot samples

The spot samples provided 762 measurements of urine osmolality and creatinine. The water intake per 24 h was 2,627 (1,017) mL during the first 3 days and 3,364 (1,079) mL during the second 3-day period when these spot samples were collected. Hence, the fluid intake was deliberately 740 mL larger (28%) during the second period. The volunteers ate 961 meals (208 consisted of only water) on these days. Each volunteer contributed a mean of 40 (11) urine specimens.

Urine osmolality and creatinine decreased gradually as more fluid was ingested, although the overlap was considerable (Table 2). No marked changes in these results occurred when Table 2 was based on the body weight-corrected intake of water instead of the crude intake of water. Table 3 shows how representative the spot samples were of the mean value of the four 24-h collections made during the preceding days.

**Table 2:** Urine osmolality and the urinary creatinine concentration measured in spot samples for increasing daily intakes of water. Data are the mean (SD)

Water intake 24 h (L)	U-osmolality (mosmol/kg)	U-creatinine (mmol/L)	Spot samples (N)	Volunteers contributing samples (N)	Sample per volunteer (N)	Meals (N)	Spot sample per meal (N)
0.9–1.5	853 (237)	14.2 (6.2)	27	4	6.8	41	0.66
1.5–2.0	582 (270)	10.1 (6.6)	79	8	9.9	101	0.78
2.0–2.5	549 (252)	9.7 (6.2)	134	12	11.2	174	0.77
2.5–3.0	545 (251)	8.5 (5.8)	203	15	13.5	258	0.79
3.0–3.5	479 (242)	7.4 (5.0)	111	13	8.5	129	0.86
3.5–5.0	390 (228)	6.7 (5.3)	141	14	10.1	181	0.78
5.0–6.9	233 (117)	3.0 (2.7)	67	10	6.7	77	0.87

**Table 3:** Comparison between the mean biomarker values at four different time periods of the day and the mean of the four 24-collections of urine performed during the preceding days. Time was missing for 3 spot samples. Data are the mean (SD)

	(1) 0–7 h	(2) 7–14 h	(3) 14–20 h	(4) 20–24 h	ANOVA *P*-value
**Normal water intake**					
Sample count	49	116	111	72	
Osmolality (mosmol/kg) Spot	529 (234)	564 (283)	557 (305)	562 (293)	0.90
Spot – 24 h	-14 (243)	+ 67 (239)	+ 46 (237)	+ 45 (212)	0.26
Creatinine (mmol/L) Spot	9.7 (5.7)	9.3 (6.7)	8.2 (6.1)	8.5 (5.7)	0.36
Spot – 24 h	+ 1.3 (5.7)	+ 2.0 (5.5)	0.0 (4.9)	+ 0.6 (3.7)	0.03 ^1^
**Increased water intake**					
Sample count	62	130	131	88	
Osmolality (mosmol/kg) Spot	519 (211)	462 (234)	419 (252)	397 (232)	0.01 ^2^
Spot – 24 h	+ 72 (214)	+ 44 (221)	-12 (208)	-48 (198)	0.02 ^3^
Creatinine (mmol/L) Spot	10.5 (6.3)	7.8 (5.5)	6.5 (5.6)	5.9 (4.7)	0.001 ^4^
Spot – 24 h	+ 3.9 (6.0)	+ 1.6 (5.3)	-0.1 (4.4)	-0.7 (4.1)	0.001 ^5^

Scheffé post hoc test shows differences by *P* < 0.05; ^1^ 3 < 2; ^2^ 4 < 1; ^3^ 4 < 1, 2; ^4^ 4 < 1, 2; ^5^ 3,4 < 1, 2

The AUC for the ROC curves that compared the osmolality of the spot urine samples with the measured 24 h water consumption during the same day averaged 0.74 during normal fluid intake and 0.64 during increased fluid intake (Fig. 3). Urine creatinine indicated the same water intake with a slightly lower AUC (Supplementary Fig. 2).

One example from the period of normal water intake is that 52 of 56 spot samples associated with a 24 h fluid intake of > 4 L were in the lowest osmolality range (50–400 mosmol/kg). Hence, low osmolality identified nearly all days with water intake > 4 L, but the sensitivity was only 43% due to frequent false indications. The specificity was still 98%, which suggests that urine osmolality > 400 mosmol/kg can preclude very large daily intakes of water (Fig. 3A).

Another example is that water intake of < 1.5 L was associated with 27 spot samples in 4 volunteers; 17 of those samples showed urine osmolality > 800 mosmol/kg. However, the urine osmolality was also this high  during many days when more  water was ingested. Sensitivity was only 21%, but the specificity 96%. Hence, a urine osmolality of < 800 seems to serve as a useful biomarker of water intake of > 1.5 L (Fig. 3C).

## Discussion

### Key results

**Fig. 2:**
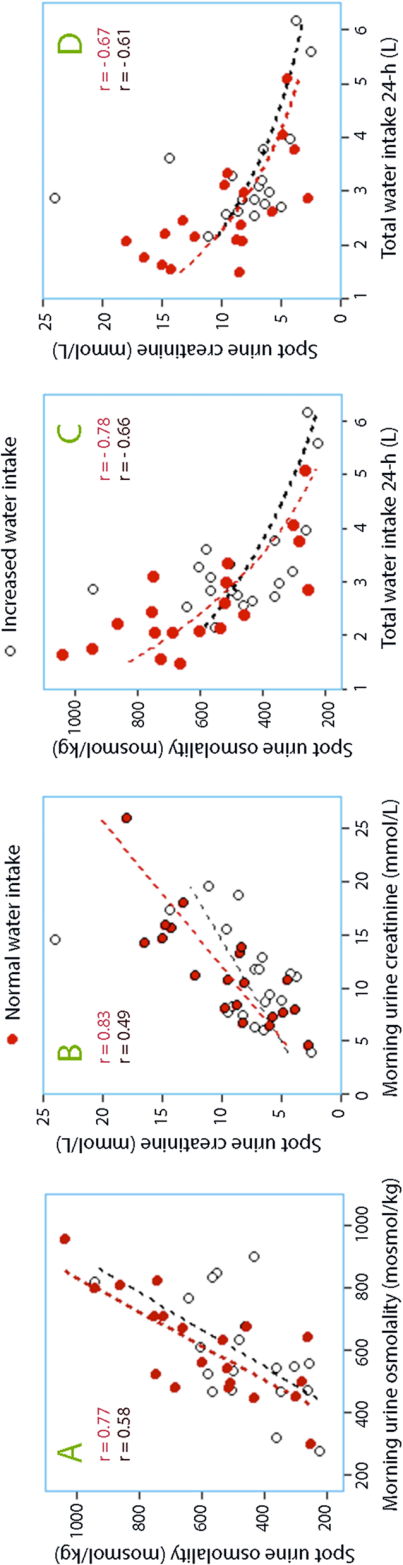
**A** Mean osmolality of the morning urine during the four days of normal intake of water (Days 1–4) and increased intake of water (Days 8–11) *versus* the mean values of the spot samples during the subsequent three days (Days 5–7 and 12–14, respectively). Each point represents one volunteer. **B** Same plot but for urine creatinine. **C** Relationship between the measured intake of water and the mean value of the spot urine osmolality samples obtained during the same 24 h. **D** Same plot but for urine creatinine

The present study demonstrates relationships between urinary biomarkers of hydration in spot samples and in 24 h collections of urine, as well as curvilinear relationships between spot samples and water intake (Fig. 2). The expected urine concentrations of the biomarkers in spot samples for increasing amounts of water consumptions are also reported (Table 2). However, the focus is placed on calculating the sensitivity the specificity for ranges of biomarkers to quantify the water intake, which is relevant information when using urine biomarkers in population studies.

The results show that morning urine and spot urine samples had almost the same ability to discriminate between ranges of daily water intakes, albeit that spot samples were slightly poorer. The osmolality and the creatinine concentration both had a reasonably good capacity to indicate large water intakes (> 3–4 L). Small volumes (< 1.5–2 L) were indicated by highly concentrated urine, with only a few misses, but the sensitivity was hampered due to many “false alarms.” The usefulness of these biomarkers was reduced when the subject was in the process of increasing the daily intake of water. One should note that the 24-h urine collection and the spot samples predicted the water intake during the same day while the morning urine summarized the water consumption for the preceding 24 h.

### Urine analysis

Urine sampling for measurement of metabolic waste products has gained recognition as a tool for monitoring body hydration in sports medicine [2–4], whereas its use in other settings, such as in population studies and geriatric hospital care, is rare [11, 12]. Uncertainty about the accuracy of urine sampling in indicating water ingestion in individuals rather than groups has certainly contributed to its limited applicability. The complexity of the issue has been further increased by different modes of sampling.

The present study offers orientating guidance on these issues. The reference data consist of careful registration of all water in liquid and food consumed during a two-week period by healthy hospital workers. The ability of two urine biomarkers — osmolality and creatinine — to indicate the water consumption was tested during periods of both normal and increased fluid intake. Several modes of sampling were used, and ROC curves were employed to study the sensitivity and specificity of each mode to detect the water ingestion in an individual.

### ROC curves

The first step was to determine how well measurements of biomarkers in a 24 h collection of urine could predict the urine volume. Both variables were known, and this comparison then served as a reference for methodological errors as well as between-subject and between-measurement variability. The AUC of the ROC curves then averaged 0.88.

The AUC decreased to 0.77 when the same biomarkers were used to discriminate between pre-set arbitrary ranges of water intake; the difference may be due to variable insensible fluid losses, different degrees of fluid retention, and inaccuracies in the quantification of water intake.

**Fig. 3:**
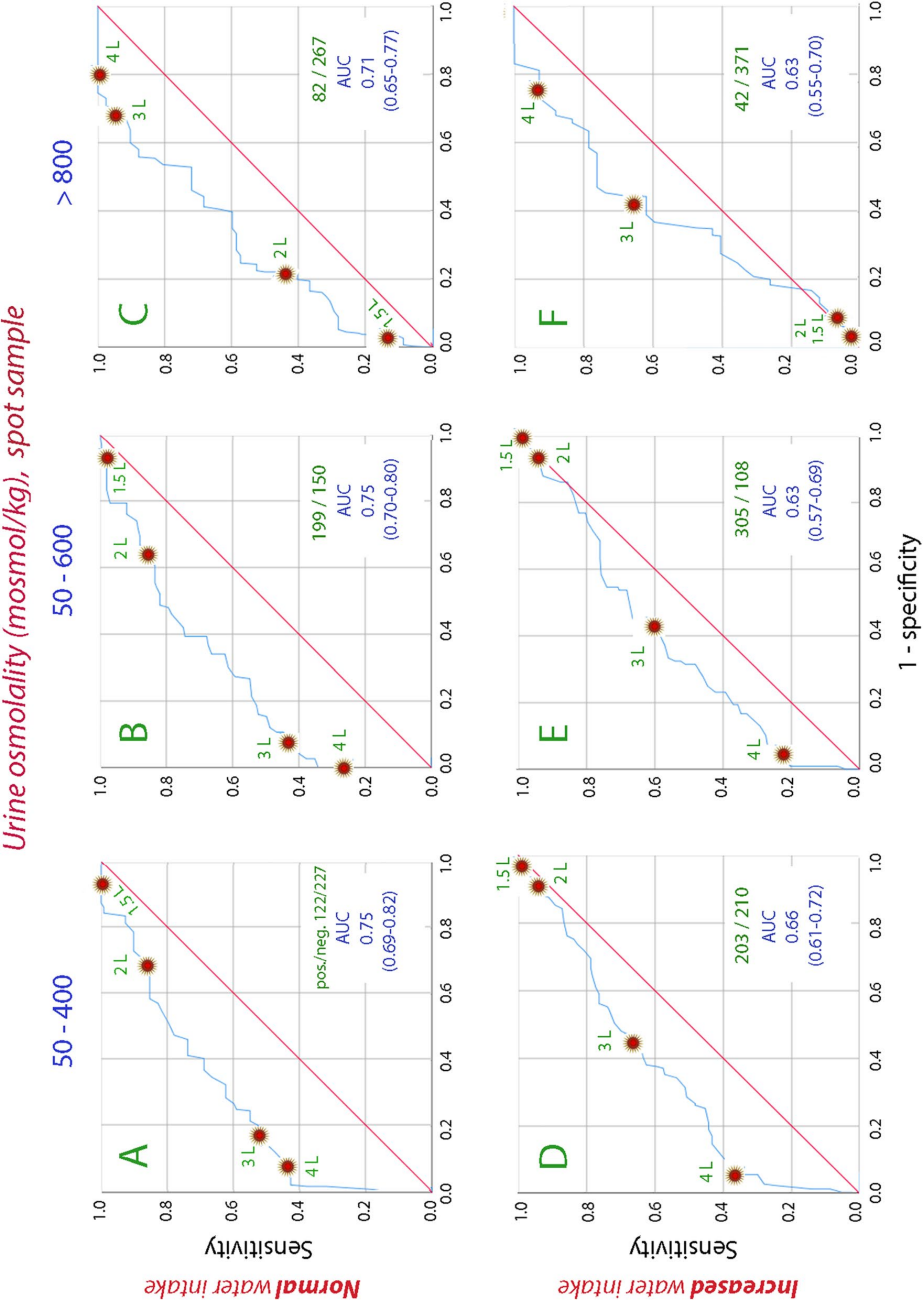
Receiver operating characteristic (ROC) curves that show the sensitivity and (1 – specificity) for ranges of urine osmolality in spot urine samples to indicate the water consumption during the preceding day. AUC = area under the curve

Morning urine showed only slightly poorer discriminating ability, but the AUC for osmolality as the biomarker for the 24 h water ingestion still averaged 0.72 (Fig. 1). Surprisingly, the AUC for spot samples, which were collected during the last 3 days of each week, showed the same AUC as the morning samples, with an average of 0.74 for the osmolality (Fig. 3).

### Habitual or increased fluid intake

It is now well established that the urine osmolality and the urinary creatinine concentration vary with the habitual intake of water in healthy individuals who do not exercise [6, 7, 10]. A methodological problem is that an increase in ingested volume of 30% was not enough to significantly increase the dilution of the morning urine [10]. The slowness of the kidneys to adapt to such a diet change is reflected by that low biomarker levels most reliably indicated large intakes of water during the first week, while high levels gave a more accurate indication during the second week.

Acute ingestion of water as well as exercise-induced dehydration make the kidneys adapt within hours [13–15] but quite large increases of the water consumption seem to be needed (> 1.5 L per day) [16, 17]. Thus, renal adaptation from conserving to excreting water occurs faster than indicated by the morning urine. For example, intravenous volume loading with 1.5 L of electrolyte-containing fluid over 30 min in elderly males showed that the initial setting of the kidneys to excrete or retain water strongly influences the excreted volume during the first hour, and that some effect remains for at least three hours [18].

### Literature

Urine analysis to indicate the water balance in male athletes was initiated in the early 1990s by Armstrong et al*.* [13]. The focus was on the visual estimation of urine color as compared to laboratory analyses of urine-specific gravity and urine osmolality [3].

Further studies in sports medicine focused on progressive dehydration and included plasma osmolality [3, 4, 14]. Cheuvront et al*.* found a sensitivity of 90% for urine osmolality and for urine-specific gravity to detect dehydration when defined as a rise in plasma osmolality from baseline [15].

Urine analysis was later extended to a wide age span and both genders during recreational sports and showed that a loss of 1% of the body weight was detectable [19]. Attention was also given to the variability in urine concentration in the population [4] and to concentrated urine as a risk factor for a poor outcome in geriatric care [11, 20, 21].

Attempts have been made to identify a fixed time of the day when measurement of a biomarker in a single urine sample could yield the same result as a 24-h collection. Two studies report that urine osmolality measured in the early or late afternoon correlate best with the osmolality of 24-h collections [7, 8]. A similar evaluation was performed in the present study but designed to show how robust spot samples are to reflect the 24-h excretion of biomarkers during four days in an individual volunteer. The results confirm that afternoon samples reflect the 24-h collection better than morning samples, at least during the period of increased water consumption (Table 3). However, the scatter of the data was still considerable, making it unclear how reliable a single spot sample is to indicate the excretion of biomarker in 24-h urine collections during a four-day period. On the other hand, a spot sample taken in the afternoon seems to be a useful indicator of hydration if larger populations are compared.

The accuracy of spot samples may be more clearly affected by the time after intake of water and food. Liberal water consumption during a meal induces acute diuresis, whereby the biomarkers in the urine become temporarily diluted. A previous report showed that spot samples were 20–30% more diluted than morning samples for 7 h after intake of food or water, while after 7 h they were 5–10% less diluted. By contrast, 24 h collections of urine for biomarker analysis yielded quite similar mean values to those of the spot samples during the first 7 h, while the spot samples taken later showed higher concentrations [10].

### Dehydration

The kidneys concentrate the urine over a very wide range [5]. This is the normal adaptation to variations in the daily consumption of water and does not implicate dehydration, as the body fluid volumes are well maintained [10]. Several studies from sports medicine show that a change in urine biomarkers from a known baseline can diagnose acute exercise-induced dehydration [15]. However, relying on a single sample for this purpose in a mixed population is futile, since the inter-individual variation in normal water ingestion is great; the reference point is unknown because the kidneys may have adapted well to a chronically low intake of water. This issue has caused distrust in the ability of urine analysis to diagnose chronic dehydration and low fluid intake [12, 22].

Biomarkers measured in the plasma, such as creatinine and osmolality, may be used to indicate dehydration, but changes develop late and only when the renal water conservation mechanisms are insufficient. Blood sampling also lacks the simplicity of the urine analysis. Hyperosmolality is often used as a sign of “intracellular” dehydration [14, 15], and this suggests that the kidneys are too slow to cope with sudden losses of water. In chronic dehydration, hyperosmolality develops when the capacity to concentrate the urine is exhausted, and this can be due to a low intake of water but also to age-related impairment of the renal concentrating capacity. Therefore, no correlation has been found between serum osmolality and single measurements of urine biomarkers of hydration in the elderly [12, 22, 23]. Chronic dehydration remains a challenging diagnosis that still needs be based on a combination of medical history, clinical signs, and measurements. None of the volunteers in the current presentation was considered dehydrated, based on the hemodynamic measurements, body fluid volumes as measured with bioimpedance, and the fact that they were full-time active hospital workers.

Visual estimation followed by manual recording of the water consumption is a commonly used alternative to urine analysis for monitoring of the fluid intake in sick patients. This approach requires much attention and involves considerable uncertainties. Ingestion of fluid is sometimes simply missed. Moreover, patients visually underestimate, and healthy volunteers commonly overestimate, the amount of fluid that is contained in a glass, while nurses make the best estimates [24]. Lack of knowledge about how to judge the water content of solid food further reduces the value of visual estimates of fluid intake.

Tucker et al*.* correlated the void frequency with the degree of hydration in male volunteers [25]. They voided 7 (2) times per 24 h during habitual fluid intake (mean, 2.4 L) and 5 (2) times when being hypohydrated (intake 1 L per 24 h). The void frequency was considered a reliable indicator of hydration status despite the apparent overlapping indicated by the two standard deviations. In the present study, the void frequency increased stepwise with the fluid intake up to 3 L per 24 h (Table 2). However, counting the void frequency requires monitoring over 24 h and the idea of relying on a single measurement of a biomarker is lost.

### Practical application

No urine analysis provides precise information about the 24 h fluid intake. Any judgment of what represents “good” and “poor” sensitivity and specificity in this setting is up to the reader. The following conclusions represent the views of the author.

High water intakes can be indicated in both morning and spot samples that show osmolality and creatinine in the lowest range (50–400 mosmol/kg and 2–6 mmol/L, respectively). False indications are rare.

The most important information is whether a subject ingests normal (2–3 L) or small (< 1.5–2 L) amounts of water. Urine osmolality in the highest range indicates low fluid intakes with few misses, but false indications (high osmolality but large fluid intake) are common. Therefore, diagnosis should be corroborated by other measurements.

The present results suggest that an investigator should  ascertain that a marked change in the fluid intake has not occurred during at least one week preceding the urine sampling.

### Strengths and limitations

The strengths of the study include that that *all* water intake was recorded, i.e., even the water that was contained in food. The volunteers were hospital workers, usually nurses, with experience in following a protocol and reporting. Collection of data was performed in a standardized way. Biochemical analysis was performed within 36 h, even on weekends, by an accredited hospital laboratory. Medical checkups were performed on three occasions during the 2-week study, ensuring hemodynamic stability and that no signs of dehydration or disease appeared [10].

Limitations include a small number of subjects, although each volunteer contributed with 8 morning urine samples that were matched with the same number of 24 h measurements of water intake. The number of spot samples was 10 times higher, which means that several spot samples were associated with the same calculation of the 24 h water intake. However, different data were used for the morning urine and spot sample analyses, which complicates direct comparisons.

As evident from Table 2, the distributions of spot sample data across ranges of water intakes were not balanced. For example, only 4 volunteers contributed the 27 samples associated with fluid intakes < 1.5 L, all of which occurred during normal fluid intake.

Four arbitrary ranges of biomarkers were used. Only three are shown in the figures, but the fourth (osmolality 600–800 mosmol/kg and urine creatinine 9–15 mmol/L) can be inferred from the others.

The indications are not valid in association with strenuous sport activities or profuse sweating.

## Conclusions

A 2-week study in 20 volunteers outlined how well habitual and increased water intake can be indicated by biochemical analysis of osmolality and creatinine in morning and spot sample urine. ROC curves suggested that the ability of ranges of biomarkers to discriminate between ranges of water intake is similar for morning and spot sample urine. However, the analyses only demonstrate reasonably good separation of small from very large intakes of water. The ability of the biomarkers to indicate water consumption was poorer when the intake was deliberately increased.

## Supplementary Information

**Additional file 1: Supplementary figure 1.** Receiver operating characteristic (ROC) curves that show the sensitivity and (1 – specificity) for ranges of urine creatinine in the morning urine to indicate the water consumption during the preceding day. AUC = area under the curve. **Supplementary figure 2.** Receiver operating characteristic (ROC) curves that show the sensitivity and (1 – specificity) for ranges of urine creatinine in spot urine samples to indicate the water consumption during the preceding day. AUC = area under the curve.

**Additional file 2. Data used to analyze the morning urine.**

**Additional file 3. Data used to analyze the spot urine.**

**Additional file 4. All original data.**

## Data Availability

The morning urine data is available as Supplementary File 1.xls and the spot sampling data as Supplementary File 2.xls.

## Abbreviations

ANOVA: Analysis of variance
AUC: Area under the curve
r: Correlation coefficient
ROC: Receiver operating characteristic

## References

[1] Macdiarmid J, Blundell J (1998). Assessing dietary intake: who, what and why of under-reporting. Nutr Res Rev.

[2] Armstrong LE, Soto JA, Hacker FT, Casa DJ, Kavouras SA, Maresh CM (1998). Urinary indices during dehydration, exercise and rehydration. Int J Sport Nutr.

[3] Popowski LA, Oppliger RA, Lambert GP, Johnson RF, Johnson AK, Gisolf CV (2001). Blood and urinary measures of hydration status during progressive acute dehydration. Med Sci Sports Exerc.

[4] Casa DJ, Armstrong LE, Hillman SK, Montain SJ, Reiff RV, Rich BS (2000). National athletic trainers’ association position statement: Fluid replacement for athletes. J Athl Train.

[5] Hahn RG, Grankvist N, Krizhanovskii C (2016). Urinary analysis of fluid retention in the general population: a cross-sectional study. PLoS ONE.

[6] Perrier E, Vergne S, Klein A, Poupin M, Rondeau P, Le Bellego L, Armstrong LE, Lang F, Stookey J, Tack I (2013). Hydration biomarkers in free-living adults with different levels of habitual fluid consumption. Br J Nutr.

[7] Perrier E, Demazières A, Girard N, Pross N, Osbild D, Metzger D (2013). Circadian variation and responsiveness of hydration biomarkers to changes in daily water intake. Eur J Appl Physiol.

[8] Bottin JH, Lemetais G, Poupin M, Jimenez L, Perrier ET (2016). Equivalence of afternoon spot and 24-h urinary hydration biomarkers in free-living healthy adults. Eur J Clin Nutr.

[9] Cheuvront SN, Kenefick RW, Zambraski EJ (2015). Spot urine concentrations should not be used for hydration assessment: a methodology review. Int J Sport Nutr Exerc Metab.

[10] Hahn RG (2021). Effects of diet, habitual water intake and increased hydration on body fluid volumes and urinary analysis of renal fluid retention in healthy volunteers. Eur J Nutr.

[11] Johnson P, Waldreus N, Hahn RG, Stenström H, Sjöstrand F (2015). Fluid retention index predicts the 30-day mortality in geriatric care. Scand J Clin Lab Invest.

[12] Johnson P, Hahn RG (2018). Signs of dehydration in nursing home residents. JAMDA.

[13] Armstrong LE, Maresh CM, Castellani JW, Bergeron MF, Kenefick RW, LaGasse KE (1994). Urinary indices of hydration status. Int J Sport Nutr.

[14] Oppliger RA, Magnes SA, Popowski LA, Gisolfi CV (2005). Accuracy of urine specific gravity and osmolality as indicators of hydration status. Int J Sport Nutr Exerc Metab.

[15] Cheuvront SN, Ely BR, Kenefick RW, Sawka MN (2010). Biological variation and diagnostic accuracy of dehydration assessment markers. Am J Clin Nutr.

[16] Johnson EC, Munoz CX, Bellegro L, Klein A, Casa DJ, Maresh CM (2015). Markers of the hydration process during volume modification with habitual high or low daily fluid intakes. Eur J Appl Physiol.

[17] Johnson E, Huffman AE, Yoder H, Dolci A, Perrier ET, Larson-Meyer ED, Armstrong LE (2020). Urinary markers of hydration during 3-day water restriction and graded rehydration. Eur J Nutr.

[18] Hahn RG (2021). Renal water conservation and the volume kinetics of fluid-induced diuresis; a retrospective analysis of two cohorts of elderly men. Clin Exp Pharm Physiol.

[19] Hahn RG, Waldréus N (2013). An aggregate urine analysis tool to detect acute dehydration. Int J Sport Nutr Exerc Metab.

[20] Ylinenvaara SI, Elisson O, Berg K, Zdolsek JH, Krook H, Hahn RG. Preoperative urine-specific gravity and the incidence of complications after hip fracture surgery. A prospective, observational study. Eur J Anaesthesiol. 2014;31:85–90. 10.1097/01.EJA.0000435057.72303.0e 24145802

[21] Hahn RG (2017). Renal water conservation determines the increase in body weight after surgery; a randomized controlled trial. Saudi J Anaesth.

[22] Hooper L, Bunn DK, Abdelhamid A, Gillings R, Jennings A, Maas K (2016). Water-loss (intracellular) dehydration assessed using urinary tests: how well do they work? Diagnostic accuracy in older people. Am J Clin Nutr.

[23] Ekman L, Johnson P, Hahn RG (2020). Signs of dehydration after hip fracture surgery: an observational descriptive study. Medicina.

[24] Frydenlund Michelsen C, Søndergaard Svendsen MB, Lommer Bagger M, Konradsen H (2022). A study on accuracy and precision of fluid volume measurements by patients, nurses, and healthy persons. Nurs Open.

[25] Tucker MA, Gonzalez MA, Adams JD, Burchfield JM, Moyen NE, Robinson FB (2016). Reliability of 24-h void frequency as an index of hydration status when euhydrated and hypohydrated. Eur J Clin Nutr.

