# Supplementary material for: Quantifying the daily intake of water from morning and spot urine samples; retrospective analysis of a clinical trial in volunteers
Source: BMC Nutr. 2023 Jan 2;9:3. doi: 10.1186/s40795-022-00660-2 (PMC9809043; doi:10.1186/s40795-022-00660-2)
Supplement: Supplementary file 1 — Additional file 1: Supplementary figure 1. Receiver operating characteristic (ROC) curves that show the sensitivity and (1 – specificity) for ranges of urine creatinine in the morning urine to indicate the water consumption during the preceding day. AUC = area under the curve. Supplementary figure 2. Receiver operating characteristic (ROC) curves that show the sensitivity and (1 – specificity) for ranges of urine creatinine in spot urine samples to indicate the water consumption during the preceding day. AUC = area under the curve. [file 40795_2022_660_MOESM1_ESM.docx]

**Supplementary figures 1 and 2**

**
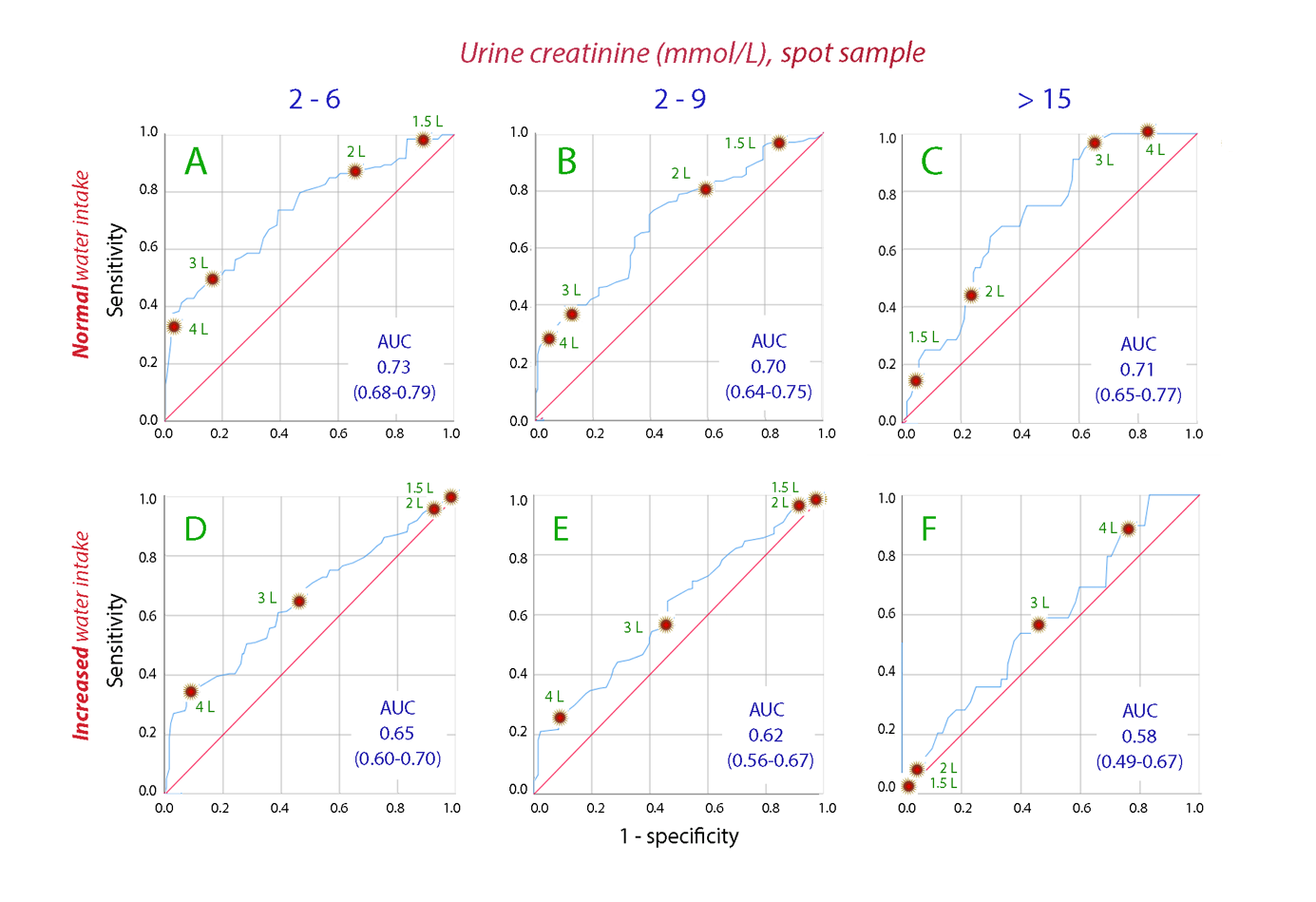
**

**Supplementary figure 1**

Receiver operating characteristic (ROC) curves that show the sensitivity and (1 – specificity) for ranges of urine creatinine in the morning urine to indicate the water consumption during the preceding day. AUC = area under the curve.

**
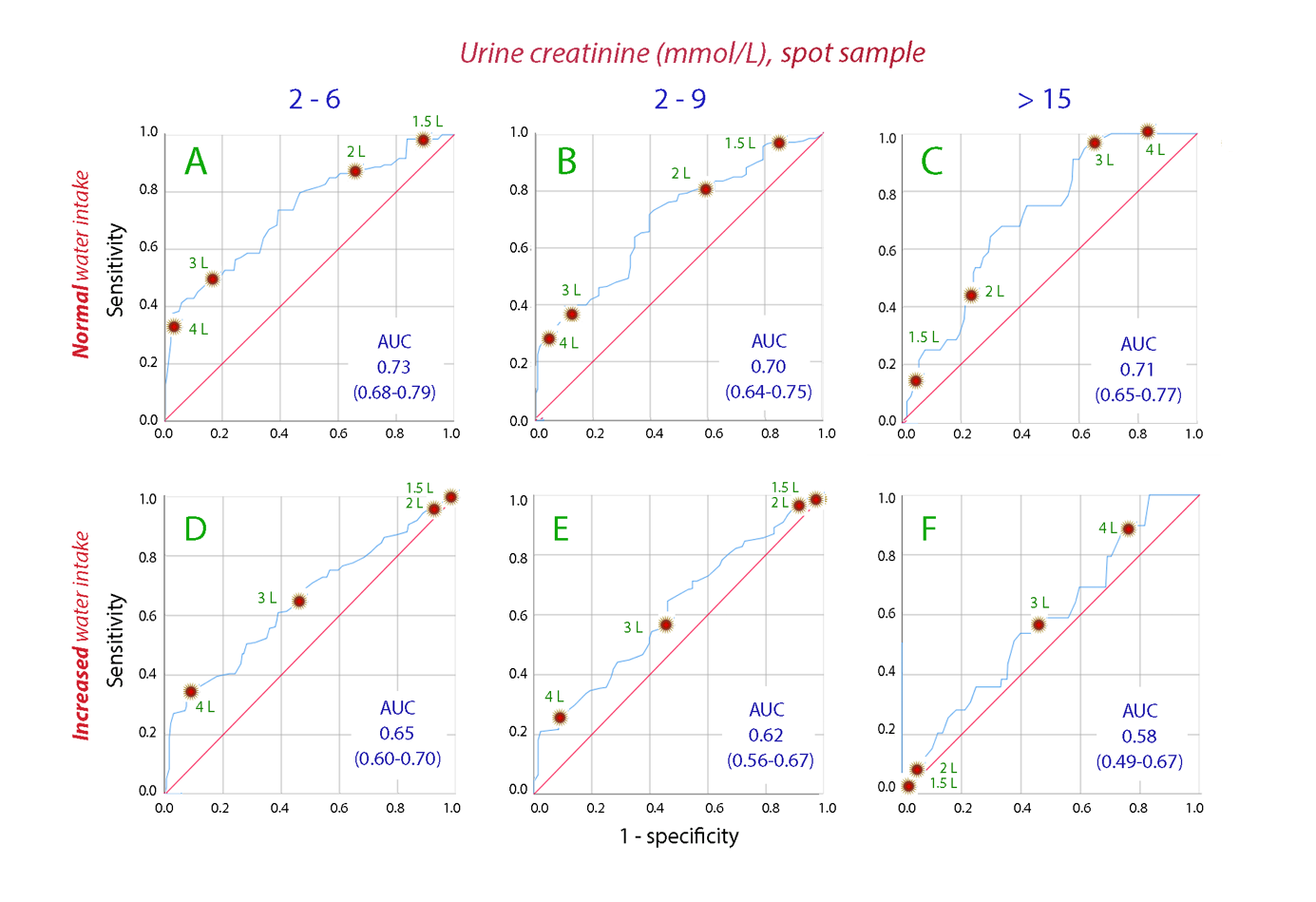
**

**Supplementary figure 2**

Receiver operating characteristic (ROC) curves that show the sensitivity and (1 – specificity) for ranges of urine creatinine in spot urine samples to indicate the water consumption during the preceding day. AUC = area under the curve.
